# Diagnostic assessment of patients with Meniere's disease through caloric testing and the video-head-impulse test

**DOI:** 10.1016/j.bjorl.2019.10.008

**Published:** 2019-11-22

**Authors:** Lívia Noleto de Rezende Oliveira, Cleydson Lucena de Andrada Oliveira, Karen de Carvalho Lopes, Fernando Freitas Ganança

**Affiliations:** Universidade Federal de São Paulo (UNIFESP), Escola Paulista de Medicina, Disciplina de Otologia e Otoneurologia, São Paulo, SP, Brazil

**Keywords:** Meniere's disease, Vertigo, Hearing loss, Vestibular functional test

## Abstract

**Introduction:**

Meniere's disease is a labyrinth disease that usually presents with episodes of spontaneous vertigo associated with sensorineural hearing loss, tinnitus and ipsi- and unilateral aural fullness in most cases. Vestibular function tests, video-head-impulse test and the caloric test, are not specific for diagnosis of the disease, but may show alterations that help to evaluate the functional impairment.

**Objective:**

To describe the results obtained at the caloric test and video-head-impulse test in patients with definite Meniere's disease and compare them between symptomatic, asymptomatic ears and those of the control group.

**Methods:**

Cross-sectional and observational study including patients with definite Meniere's disease diagnosed according to the Bárány Society criteria (2015) and healthy individuals (control group) undergoing caloric test and video-head-impulse test. All subjects were assessed by neurotological anamnesis and audiological evaluation (pure-tone, vocal and immittance audiometry) to characterize the sample. The findings obtained at the caloric test and video-head-impulse test were described and compared between the symptomatic and asymptomatic ears of patients with Meniere's disease and those of the control group.

**Results:**

Thirty-two patients with definite Meniere's disease were evaluated, with a mean age of 45.7 years, mostly females (68.8%) and unilateral disease. The control group consisted of 20 healthy individuals, with a mean age of 44.7 years, mostly females (70.0%). The groups were homogeneous in relation to age and gender. The patients’ main complaint was vertigo (71.9%), and most patients had more than six episodes in the last six months (71.9%). Moderate sensorineural hearing loss was present in 38.5% of patients. The prevalence of hyporeflexia at the caloric test was higher in symptomatic (56.4%) and asymptomatic (36%) ears of patients with Meniere's disease compared to the ears of control subjects (7.5%), *p* < 0.001 and *p* = 0.004, respectively. Video-head-impulse test alterations in the lateral semicircular canals were more frequent in the symptomatic ears of patients with Meniere's disease than in the ears of control subjects (*p* = 0.026).

**Conclusion:**

Most patients with definite Meniere's disease showed hyporeflexia at the caloric test and video-head-impulse test with normal function in the symptomatic ear. Vestibular hyporeflexia at the caloric test was more frequent in the symptomatic and asymptomatic ears of patients with Meniere's disease than in the control group. The video-head-impulse test showed more alterations in the lateral semicircular canals.

## Introduction

Meniere's Disease (MD) is a clinical syndrome consisting of spontaneous vertigo episodes associated with sensorineural hearing loss (SNHL) and fluctuating hearing symptoms (hearing, tinnitus and aural fullness) in the affected ear.^1^

The histopathological substrate of MD corresponds to endolymphatic hydrops (EH), described in 1938 by Hallpike and Cairns and detectable in pathological studies of temporal bones.^2^, ^3^ EH is the excess of endolymph in the membranous labyrinth, which leads to the dilation of the cochlear duct, saccule, semicircular canals, and utricle.

Although there are no specific findings for the diagnosis of MD in vestibular tests, functional assessment of inner ear structures is important to measure disease impairment, in order to establish prognosis and more specific treatment.

The caloric test (CT) is the oldest method for functional assessment of the vestibular system, although it only evaluates the lateral semicircular canals (SCCs), through low frequency stimuli, around 0.002–0.004 Hz, allowing the separate identification of the impaired labyrinth.^4^ There is no pathognomonic finding of MD at the CT, which may show normal responses, hyperreflexia, hyporeflexia, unilateral or bilateral arreflexia, although the most common result is hyporeflexia of the affected labyrinth.^5^

The video-head-impulse test (vHIT) evaluates the three SCCs on each side of the labyrinth. vHIT is the computerized quantitative analysis of the head-impulse test (HIT), which measures the angular gain of the Vestibulo-Ocular Reflex (VOR).^6^ The HIT is a very specific test of the SCC function, as its response is very fast. While stimulation is performed at low frequencies in the CT, labyrinth stimulation is performed at high frequencies in the vHIT, at around 5–7 Hz.^7^

Despite the several scientific studies carried out on MD, it is not yet clear how vestibular function is influenced by this disease. Considering the fact that it is a disease with clinical variants, a more detailed vestibular evaluation is extremely important. Further studies involving both tests are needed to provide a more accurate investigation and better follow-up for patients suffering from the disease.

The aim of this study was to describe the results obtained at the CT and vHIT in patients with definite MD and to compare the results obtained between symptomatic and asymptomatic ears of patients with MD and also with those of the control group.

## Methods

An observational and cross-sectional study was carried out at the Neurotology Outpatient Clinic of the Otology and Neurotology Discipline of the Department of Otorhinolaryngology and Head and Neck Surgery of Universidade Federal de São Paulo.

This study was approved in 2016 by the Research Ethics Committee (REC) of the Institution, Plataforma Brasil – number 59556216.8.0000.5505.

Patients of both genders aged 18–65 years old, with a definite clinical diagnosis of MD, according to the Bárány Society criteria (2015), were consecutively recruited at the Neurotology Outpatient Clinic from April 1, 2017 to December 1, 2018.^1^

Exclusion criteria included patients with other vestibular diseases, chronic otological, cervical, and eye diseases that prevented proper visualization of the target during vHIT or eye movements, diseases of the central nervous system and those who had already been submitted to an invasive procedure in any of the ears.

The control group consisted of individuals of both genders, aged between 18 and 65 years, with no auditory-vestibular complaints or other comorbidities and who agreed to participate in the study.

The patients underwent a diagnostic routine consisting of complete neurotological history, otorhinolaryngological and neurotological exams, and audiological evaluation (pure-tone, vocal and immittance audiometry). Vestibular evaluation was performed by videonystagmography (VNG) with CT and vHIT. These two tests were performed 1 h apart and at least 72 h after the MD crisis and the first exam was vHIT, followed by CT. Both examinations were performed by the same researcher, who is an otorhinolaryngologist and the main author of this study, experienced in performing these tests, and who knew which study group each subject belonged to.

Patients were instructed not to consume alcoholic or caffeinated beverages, chocolate, avoid smoking or use medications such as painkillers, vestibular suppressants (anti-vertigo and sedative drugs) for 72 h before the vestibular function tests.

The clinical questionnaire consisted of sociodemographic and clinical data, such as the disease duration, the frequency of vertigo episodes in the last six months, disease laterality and the main symptom, i.e., the one causing the most discomfort at the time of the evaluation.

The patients were distributed according to the number of vertigo episodes in the last six months into three groups: I (≤2 episodes), II (3–5 episodes), III (≥6) and according to the audiometric staging system proposed by AAO-HNS (1995), which is based on the average of the pure-tone thresholds of the 500 Hz, 1 kHz, 2 kHz and 3 kHz frequencies, the patients’ ears were classified in four stages: I (≤25 dBHL), II (26–40 dBHL), III (41–69 dBHL) and IV (≥70 dBHL).^8^

The CT was performed with the patient wearing the closed VNG mask, in the horizontal supine position, with the head extended at 30°, verticalizing the lateral channels, with the ampules in the upward position, using the air otocalorimeter (ICS Air Cal, GN-Otometrics, Denmark) (24 °C for cold testing and 50 °C for hot testing), with a volume of 8 L of air per minute and duration of 60 s, with an interval between irrigations of 5 min. After the irrigation, we waited for the Slow Component Angular Velocity (SCAV); the patient then fixed their eyes on a bright spot, which was recorded from the beginning and for another 30 s after ocular fixation. The patient performed mental activity throughout and after irrigation to decrease cortical inhibition of the post-caloric response. The stimulation sequence followed the sequence: cold right, cold left, hot left and hot right. Absolute unilateral hyporeflexia was considered when the sum of the SCAV values of the cold and hot tests, of the right or left ear was <5°/s. Absolute bilateral hyporeflexia was considered when the sum of the SCAV values in the four tests was <12°/s. To evaluate the Labyrinthine Predominance (LP), we calculated the percentage difference between the responses of the two labyrinths, quantified according to Jongkees’ formula, where the best response of the angular velocity of the slow component at each temperature and in each ear is chosen. Relative hyporeflexia was considered in cases when LP was >19%.^9^

To perform v-HIT (ICS, GN Otometrics, Denmark), unpredictable manual movements were performed on the yaw axis for lateral canal testing. The impulses must have an angle between 15° and 20° from midline, velocity between 100° and 250°/s and acceleration between 1000° and 2500°/s^2^. The vertical canal tests were performed in the diagonal plane, between the roll and pitch axes, with the head rotated 35° to the right or left from midline.

The test plane when the head is rotated to the right takes the LARP (Left Anterior and Right Posterior) plane; when turned 35° to the left, it assumes the RALP (Right Anterior and Left Posterior) plane. These movements can have an amplitude between 10° and 20°, velocity between 80° and 250°/s and acceleration between 750° and 5000°/s^2^.^7^, ^10^

At least 20 stimuli were obtained in each movement plane. The sensors detect eye and head movements and transcribe them into a chart visible on the computer screen.

When the chart of eye and head movements is similar, the computer program accepts the situation as normal gain and close to 1.00. However, during a cephalic impulse, if eye movement is less than necessary to keep the gaze fixed on the target, the gain will be less than 1.00 and a second eye movement occurs, the corrective saccade. The equipment considers a decrease of up to 0.20 for the lateral SCCs and 0.25 for the vertical ones as normal, due to its precision characteristics.

An altered vHIT was considered in the presence of decreased gain and of corrective saccades after the head movement.

Data were stored and tabulated using Microsoft Excel 2011® program by the researcher. Initially, all variables were analyzed descriptively. The significance level used for the tests was set at 5%.

## Results

The group of patients with MD consisted of 22 women (68.8%) and 10 men (31.2%), aged between 18 and 62 years (mean = 45.7). The control group consisted of 20 individuals, 14 (70.0%) women and 6 (30.0%) men, aged 28–62 years (mean = 44.7). The groups showed homogeneity regarding age and gender.

According to the patients’ clinical characteristics, the mean disease duration was 3 years, with a minimum duration of 3 months and a maximum of 42 years. Regarding disease laterality, 13 patients (40.6%) showed involvement of the right ear, 12 patients (37.5%) of the left ear and 7 patients (21.9%) had bilateral involvement.

As for the MD classification, according to the number of episodes in the previous 6 months, 28.1% reported up to two episodes in the previous 6 months and 71.9% reported more than six episodes in that period. The stage of hearing loss observed in most patients was Type III (33.3%), which represented moderate sensorineural hearing loss, according to the AAO-HNS criteria. As for the major discomfort symptom, 68.8% of the patients reported vertigo, 28.1% reported tinnitus and 3.1% reported hearing loss.

Regarding the prevalence of absolute or CT-related hyporeflexia, in the symptomatic, asymptomatic ears and control group it was 22 (56.4%); 9 (36%) and 3 (7.5%), respectively. The symptomatic and asymptomatic ears showed a higher occurrence of absolute or relative hyporeflexia compared to the control (Table 1).

**Table 1:** Prevalence of absolute or relative hyporeflexia at the caloric test in Meniere's disease patients and control subjects.

CT	Symptomatic ear (*n* = 39)	Asymptomatic ear (*n* = 25)	Control (*n* = 40)	*p*-value
Hyporeflexia	22 (56.4%)	9 (36%)	3 (7.5%)	
*p*-value_(Sympt.×control)_	–	–	–	<0.001
*p*-value_(Assympt.×control)_	–	–	–	=0.004
*p*-value(_Sympt.×Assympt.)_	–	–	–	=0.111

CT, Caloric Test; Sympt., symptomatic; Assympt., asymptomatic.

The frequency of alterations in the vHIT, when considering the decreased gain and presence of covert and/or overt saccades, was more frequent in the lateral SCCs in symptomatic ears when compared to the control group, with a statistically significant difference (Table 2).

**Table 2:** Frequency of alterations at the vHIT when considering decreased vestibular–ocular reflex gain and presence of saccades in the evaluation of lateral, anterior and posterior semicircular canals in patients with Meniere's Disease and in the control group individuals.

SCCs	Symptomatic ear	Asymptomatic ear	Control	*p*-value
Lateral	5 (12.8%)	1 (4.0%)	0 (0.0%)	
*p*-value_(Sympt.×control)_	–	–	–	0.026
*p*-value_(Assympt.×control)_	–	–	–	0.385
*p*-value(_Sympt.×Assympt.)_	–	–	–	0.391
Anterior	2 (5.1%)	0 (0.0%)	0 (0.0%)	–
*p*-value_(Sympt.×control)_	–	–	–	0.241
*p*-value_(Assympt.×control)_	–	–	–	1.000
*p*-value(_Sympt.×Assympt.)_	–	–	–	0.516
Posterior	3 (7.7%)	0 (0.0%)	0 (0.0%)	
*p*-value_(Sympt.×control)_	–	–	–	0.116
*p*-value_(Assympt.×control)_	–	–	–	1.000
*p*-value(_Sympt.×Assympt.)_	–	–	–	0.275

SCCs, semicircular canals.

Most patients with MD showed no alterations when submitted to the vHIT. The symptomatic ears that had altered vHIT also showed CT with hyporeflexia (Table 3).

**Table 3:** Clinical presentation of absolute or caloric test-related hyporeflexia in symptomatic ears in the presence or absence of alterations in the video-head-impulse test.

Symptomatic ear	vHIT with alteration	vHIT with no alteration
CT with hyporeflexia	5 (12.8%)	17 (43.6%)
CT with no hyporeflexia	0 (0.0%)	17 (43.6%)

vHIT, video-head-impulse test; CT, Caloric Test.

## Discussion

The study group included a predominance of women with a mean age of 45.7 years, unilateral involvement and no preference for laterality. These data are consistent with other studies, which found a higher prevalence of women over 40 years and unilateral disease presentation.^11^, ^12^

The symptom that caused the greatest discomfort in patients with MD in the current study was vertigo. Most patients reported more than six episodes of vertigo in the previous six months, with a negative impact of the disease on quality of life. According to Neuhauser et al., vertigo of vestibular origin accounts for 41% of sick leave cases in Germany. Vertigo patients have low performance, interruption of daily activities and avoid leaving home.^13^, ^14^

Regarding SNHL, moderate hearing loss was present in 38.5% of patients in the current sample. The hearing is usually fluctuating in the early years and reversible after episodes of vertigo. However, over time, hearing loss becomes permanent, moderate to severe.^15^, ^16^

In the CT analysis, the current study showed that patients with MD have a higher occurrence of absolute or relative hyporeflexia in the symptomatic and asymptomatic ears, when compared to the controls. The prevalence of hyporeflexia in the symptomatic ear was in agreement with Proctor. The current sample also showed a prevalence of 36% of hyporeflexia in asymptomatic ears, which differs from that found by this author, in which only 19% of patients with unilateral MD had hyporeflexia in the asymptomatic side.^17^ This can occur due to the fact that most patients have bilateral hydrops, but do not always manifest MD or they do not have the audiological criteria to define the condition as MD.^18^ Another explanation for hyporeflexia in the asymptomatic ear of MD patients would be the decrease in the contralateral SCAV, which may be part of a vestibular compensation mechanism.^19^

Regarding the vHIT, in the current study, there was a higher prevalence of vHIT alterations in lateral SCCs in the symptomatic ears of patients with MD when compared to the control group. This fact may be attributed to a higher technical sensitivity of the lateral canal tests than the vertical ones. In contrast, Fukushima et al. showed that alterations in the vHIT was more frequently observed in the posterior SCCs, followed by the lateral ones.^20^

It was observed that hyporeflexia in the CT occurred in more than 50% of the patients; however, most vHIT results were within the normal range. The alterations in the CT are consistent with the study by Blodow et al., who found altered CT in 67% of patients with MD, whereas the vHIT showed alterations in 37% of the cases, a higher percentage than that found in the current study, which was 12%. However, in that study, unlike the present one, the inclusion criteria did not specify whether the patients had probable and/or definite MD.^21^ Another study by Rubin et al. found an altered CT in 94% and a normal vHIT in 100% of patients with definite MD; however, all patients were at the advanced stage of the disease.^22^

In the present sample, all ears that showed altered vHIT also had hyporeflexia at the CT. Mahringer et al. performed a study comparing CT and vHIT in patients with complaints of vertigo or dizziness in a community hospital. These patients had different clinical diagnoses of vestibular diseases and 15% of these had MD. The authors considered only the patients that had hyporeflexia at the CT, of which 41% also had vHIT alterations.^23^ According to Rambold, although vHIT is a time-saving test that optimizes the work, in patients with MD, the CT is more efficient for diagnosing vestibular function alterations.^24^

The VHIT and CT measure different aspects of VOR.^25^, ^26^ As with the CT, the vHIT also showed no pathognomonic signs in MD. However, unlike that observed at the CT, there was no significant difference in vHIT results between patients’ and controls’ ears. This difference between the results of both vestibular tests can be explained by the neurological semiology of the semicircular canal ampullary crest, in which MD preferentially damages Type II cells located on the organ periphery, while Type I cells located in the central region, are usually spared. The vHIT preferentially stimulates Type I cells, while the CT stimulates Type II cells.^27^, ^28^ Another explanation for this difference is due to the fact that in EH, there is an increase in the diameter of the SCCs which, in turn, may result in lower pressure induced through the cupula during caloric stimulation, thus causing hyporeflexia. However, the increase in the diameter of the SCCs would have little effect on the vHIT.^29^ Although several studies show a higher prevalence of CT alterations when compared to the vHIT, this test cannot be considered as the gold standard for the evaluation of vestibular function alterations. The authors stressed the fact that both tests are complementary to each other.^21^, ^23^, ^24^ According to a study by Hannigan et al., altered CT with normal vHIT of the horizontal canals is more commonly associated with MD and may function as a diagnostic marker of the disease.^30^

This research confirms the need for the evaluation of both ears, even in cases of unilateral disease, showing that functional impairment may be present before the clinical manifestation of symptoms. Similarly, it emphasizes the importance of global assessment of all sensory structures of the inner ear.

## Conclusion

Most patients with definite MD had hyporeflexia at the CT in the symptomatic ear and no abnormalities at the vHIT.

Absolute or relative hyporeflexia was more frequent in symptomatic and asymptomatic ears of patients with definite MD when compared to controls. Alterations in the vHIT were more frequent in the lateral canals of symptomatic ears of patients with MD.

## Conflicts of interest

The authors declare no conflicts of interest.
